# Efficacy, safety, and immunogenicity of the *Shigella sonnei* 1790GAHB GMMA candidate vaccine: Results from a phase 2b randomized, placebo-controlled challenge study in adults

**DOI:** 10.1016/j.eclinm.2021.101076

**Published:** 2021-08-13

**Authors:** Robert W. Frenck, Valentino Conti, Pietro Ferruzzi, Augustin G.W. Ndiaye, Susan Parker, Monica Malone McNeal, Michelle Dickey, Juan Paolo Granada, Giulia Luna Cilio, Iris De Ryck, Francesca Necchi, Akamol E. Suvarnapunya, Omar Rossi, Alessandra Acquaviva, Lakshmi Chandrasekaran, Kristen A. Clarkson, Joachim Auerbach, Elisa Marchetti, Robert W. Kaminski, Francesca Micoli, Rino Rappuoli, Allan Saul, Laura B. Martin, Audino Podda

**Affiliations:** a Division of Infectious Diseases, Cincinnati Children's Hospital Medical Center, Cincinnati, Ohio, United States; b GSK Vaccines Institute for Global Health, Siena, Italy; c Department of Pediatrics, University of Cincinnati College of Medicine, Cincinnati, Ohio, United States; d GSK, Siena, Italy; e Department of Diarrheal Disease Research, Bacterial Diseases Branch, Walter Reed Army Institute of Research, Silver Spring, Maryland, United States

**Keywords:** *Shigella sonnei*, 1790GAHB vaccine, GMMA, Human challenge study, Controlled human infection model, Shigellosis

## Abstract

**Background:**

Shigellosis accounts for substantial morbidity and mortality worldwide and is the second most common cause of moderate and severe diarrhoea in children.

**Methods:**

This phase 2b study (NCT03527173), conducted between August 2018 and November 2019, evaluated vaccine efficacy (VE), safety, and immunogenicity of a *Shigella sonnei* GMMA candidate vaccine (1790GAHB) in adults, using a *S. sonnei* 53 G controlled human infection model. Participants (randomized 1:1) received two doses of 1790GAHB or placebo (GAHB-Placebo), at day (D) 1 and D29, and an oral challenge of *S. sonnei* 53 G at D57. VE was evaluated using several endpoints, reflecting different case definitions of shigellosis. For the primary endpoint, the success criterion was a lower limit of the 90% confidence interval >0.

**Findings:**

Thirty-six and 35 participants received 1790GAHB or placebo, respectively; 33 and 29 were challenged, 15 and 12 developed shigellosis. VE was not demonstrated for any endpoint. Adverse events were more frequent in 1790GAHB versus placebo recipients post-vaccination. Anti-*S. sonnei* lipopolysaccharide (LPS) IgG responses increased at D29 and remained stable through D57 in group 1790GAHB; no increase was shown in placebo recipients.

**Interpretation:**

1790GAHB had an acceptable safety profile and induced anti-LPS IgG responses but did not demonstrate clinical efficacy against shigellosis. Baseline/pre-challenge antibody levels were higher in participants who did not develop shigellosis post-challenge, suggesting a role of anti-LPS IgG antibodies in clinical protection, although not fully elucidated in this study. For further vaccine development an increased *S. sonnei* O-antigen content is likely needed to enhance anti-LPS immune responses.

**Funding:**

GlaxoSmithKline Biologicals SA, Bill and Melinda Gates Foundation

**Research in context**

**Evidence before this study**

A licensed vaccine against *Shigella* is not widely available. Of the candidates currently under development, one conjugate vaccine composed of the O-specific polysaccharides of *S. sonnei* and *S. flexneri* 2a has reached the stage of clinical efficacy trial. Human challenge models were used to assess vaccine efficacy in several studies; a bioconjugate vaccine against *S. flexneri* 2a (Flexyn2a) demonstrated protection against the most severe illness after challenge.

**Added value of this study**

The GMMA-based *S. sonnei* vaccine (1790GAHB) has been previously shown to induce *S. sonnei*-specific antibody responses and to have an acceptable safety profile in adults from both non-endemic and endemic settings. This study evaluates this investigational vaccine in a controlled human infection model (CHIM) and provides additional information on the efficacy, and on the magnitude and bactericidal potential of anti-*S. sonnei* lipopolysaccharide serum immunoglobulin G antibodies. CHIM trials conducted in adults from high-income countries are suboptimal to predict vaccine efficacy in the target infant population of developing countries, however, interpretation of their results may provide insights on possible approaches for demonstration of clinical efficacy during development of the vaccine, including identification of immunological readouts relevant for clinical protection.

**Implications of all the available evidence**

There are still limited data available on the immunogenicity and safety of *Shigella* vaccines currently under development, especially in young children, the age group with the highest burden of disease in *Shigella* endemic regions. Although several live-attenuated and conjugate vaccine candidates have shown promising results, none have been able to demonstrate clinical efficacy against shigellosis in young children living in *Shigella*-endemic regions. Moreover, a well-defined correlate or even a reliable surrogate of protection against shigellosis is not yet available, thus, validation of *Shigella* candidate vaccines through field efficacy studies in endemic areas remains essential.

Alt-text: Unlabelled box

## 1 Introduction

Despite the recent decrease in the global diarrhoea burden, diarrhoeal diseases continue to be a major contemporary health concern, particularly amongst children below 5 years of age living in low and middle-income countries (LMICs).[1,2] The *Shigella* genus is one of the most clinically relevant pathogens contributing to diarrhoeal deaths, although over the last three decades, decreases were observed in diarrhoea-associated mortality rates attributable to *Shigella* [3]. Data collected between 2007 and 2012 in the Global Enteric Multicenter Study follow-on (GEMS-1A), evaluating the aetiology and mortality of moderate-to-severe diarrhoea in African and Asian children below 5 years of age, showed that *Shigella* was an under-recognized cause of non-dysenteric diarrhoea and strongly associated with an increased risk of death in children 12–59 months of age [4]. In 2017, the bacterium still accounted for an estimated 237,846 deaths, of which 95,861 deaths occurred amongst children below 5 years of age – making it the second leading cause of diarrhoeal deaths in this age group after rotavirus [1, 5]. Moreover, in 2016, *Shigella* accounted for 74,402 deaths amongst individuals 70 years of age or more, thus becoming the leading cause of diarrhoeal mortality in the elderly [1]. While shigellosis is endemic in LMICs in Asia and Africa, the burden of disease is also considerable in high-income countries, where at-risk populations are children below 5 years of age, men who have sex with men, and travellers to LMICs [6].

The *Shigella* genus comprises four distinct species, of which *S. flexneri* and *S. sonnei* are the leading causes of endemic shigellosis. The global epidemiology of *Shigella* is changing constantly and an increase in *S. sonnei* infections as compared to *S. flexneri* has been observed in developing countries, as well as in regions in which water and sanitation improvements are underway [7].

Treatment of *Shigella*-associated diarrhoea often includes antibiotics. However, the rapid increase of antimicrobial resistant *Shigella* species has limited treatment options [7,8]. Prevention of shigellosis by vaccination is therefore an acute unmet medical need.

Several investigational vaccines against *Shigella* are currently in different stages of clinical development, but none have been licensed for global use [9,10]. As protection from *Shigella* infection has been shown to be serotype-specific, the polysaccharide moiety of *Shigella* termed O-antigen (OAg) is the main target of vaccine development, being an important stimulus of the host immune response [11,12]. GSK Vaccines Institute for Global Health (GVGH) developed an investigational *S. sonnei* vaccine (1790GAHB) based on Generalized Modules for Membrane Antigens (GMMA). GMMA are outer membrane particles derived from bacteria genetically modified to enhance OAg/protein ratio and reduce the reactogenicity of integral lipopolysaccharides (LPS) [13]. 1790GAHB has been previously shown to induce anti-*S. sonnei* LPS serum immunoglobulin G (IgG) antibodies while also demonstrating an acceptable safety profile in adults from both non-endemic [14] and endemic [15] settings. Moreover, an additional vaccination with 1790GAHB approximately three years after a primary series of three doses of 1790GAHB administered one month apart, elicited a significant increase in antibody levels in adult participants with undetectable anti-*S. sonnei* LPS IgG prior to primary vaccination [16]. In this study, we assessed the efficacy in preventing shigellosis, safety, and immunogenicity of two doses of 1790GAHB in North American adults, using a Controlled Human Infection Model (CHIM) developed at the Cincinnati Children's Hospital Medical centre (CCHMC), Ohio, United States [17]. A summary contextualizing the results and potential clinical relevance and impact of the research is displayed in the Plain Language Summary (**Figure S1**).

## 2 Methods

### 2.1 Study design and participants

This was a single-centre, observer-blind, randomized, placebo-controlled, phase 2b human challenge trial (NCT03527173) conducted between August 2018 and November 2019 at the CCHMC. Further details on study participants and inclusion/exclusion criteria are provided in appendix (p 1) and can also be found in the study protocol, available at https://www.gsk-studyregister.com (study ID 205626).

Participants were randomized (1:1) to receive 2 doses of either 1790GAHB (1790GAHB group) or placebo (Placebo group), 28 days apart, and were enroled in four overlapping cohorts (Cohorts 1–4) for logistical reasons at study site, maintaining the 1:1 ratio. Both 1790GAHB and placebo were administered via intramuscular route, in the upper deltoid of the non-dominant arm. The study vaccine was provided as a preservative-free formulation (single vial of 0.7 mL) containing *S. sonnei* 1790-GMMA (12 µg/mL measured by OAg and 200 µg/mL measured by protein content) adsorbed to Alhydrogel (0.7 mg Al^3+^/mL) in Tris-buffered saline. The 0.5 mL dose containing 1.5/25 μg of OAg/protein was obtained by dilution with the placebo suspension (Alhydrogel in Tris-buffered saline [0.7 mg Al^3+^/mL]), immediately prior to vaccination.

A challenge dose of *S. sonnei* was administered at Day 57, 28 days post-second vaccination. The target challenge consisted of 1500 colony forming units (CFU) of reconstituted lyophilized *S. sonnei* strain 53 G and was administered orally, after dilution of 1 mL of the inoculum in 30 mL of sterile saline solution (0.9%). The day before the challenge administration, participants were admitted to an inpatient unit where they had to stay for an 8-day period and were monitored daily. Eligibility was assessed before challenge and participants who did not meet the eligibility criteria or had taken antibiotics in the previous week were not administered the challenge agent and were discharged from the inpatient unit. Participants fasted for 90 min prior to receiving the challenge inoculum, and for an additional 90 min after challenge. To neutralize gastric acidity, participants drank a 120 mL solution of sodium bicarbonate approximately 5 min before drinking the challenge suspension. On the 5th day after challenge (or earlier in case of symptoms defined in appendix p 1), participants received 500 mg of ciprofloxacin twice daily for 3 days. Blood samples were collected at screening, pre- and 7 days post-administration of each 1790GAHB/placebo dose, as well as pre- and 7 days, 28 days, and 6 months post-administration of the challenge inoculum. Stool samples were collected daily during the inpatient stay to verify presence of *S. sonnei* by culture and quantitative Polymerase Chain Reaction (qPCR) analysis. All stool samples were visually assessed for consistency and blood presence. Samples that were loose or watery (Grade 3–5) were weighed and if there was visible blood, a hemoccult test was performed to confirm presence of blood.

Randomization was performed using an internet randomization system, with a minimization procedure [18] to ensure a 1:1 balance between treatments. The study was observer-blinded and laboratory staff in charge of testing human samples were also blinded to the treatment, participant, and visit number.

The study was conducted in accordance with all applicable regulatory requirements, International Conference on Harmonisation - Good Clinical Practice guidelines, and the Declaration of Helsinki. The protocol and study-related documents were reviewed and approved by the CCHMC institutional review board on 10 May 2018. All participants provided electronic consent through REDcap system (www.projectredcap.org), except for one who provided written consent because the REDcap system was not working.

### 2.2 Study objectives

The primary objective was to investigate the ability of two doses of the 1790GAHB investigational vaccine in healthy adults to reduce the incidence of shigellosis, as compared to placebo, according to the primary case definition (Table 1), after challenge with *S. sonnei* strain 53 G. The success criterion was defined as a lower limit (LL) of the 90% confidence interval (CI) >0 for vaccine efficacy (VE). Secondary efficacy objectives were to determine VE of 1790GAHB against shigellosis according to other case definitions and against other clinical outcomes, as defined in Table 1. All efficacy outcomes were assessed during a period starting with the challenge administration visit and lasting up to the end of the inpatient stay.

**Table 1:** Outcomes used to measure vaccine efficacy of the 1790GAHB vaccine in the study.

	Endpoint	Case definition	Note
1	Rate of shigellosis, 1st definition (primary endpoint)	Shedding* of *S. sonne*i 53 G accompanied by moderate *or* severe diarrhoea, **OR** shedding of *S. sonne*i 53 G with an oral temperature of ≥38•5 °C	
2	Rate of shigellosis, 2nd definition (CHIM expert group definition)	Severe diarrhoea, **OR** moderate diarrhoea, **OR** dysentery	Severe diarrhoea defined as [≥6 loose stools in 24 h] OR [>800 gs loose stools in 24 h]. Moderate diarrhoea defined as 4–5 loose stools in 24 h *AND* [oral temperature ≥38•0 °C *OR* ≥1 moderate constitutional/enteric symptom^a^*OR* ≥2 episodes of vomiting in 24 h]. Dysentery defined as ≥2 Grade 3, 4, or 5 loose stools with gross blood (hemoccult positive) in 24 h *AND* [oral temperature ≥38•0 °C *OR* ≥1 moderate constitutional/enteric symptom^a^*OR* ≥2 episodes of vomiting in 24 h]
3	Rate of shigellosis, 3rd definition	Severe diarrhoea, **OR** moderate diarrhoea# with fever or with one or more moderate constitutional/enteric symptoms, **OR** dysentery	Dysentery defined as ≥2 Grade 3, 4, or 5 loose stools with gross blood (hemoccult positive) in 24 h *AND* ≥1 reportable constitutional/enteric symptom^b^
4	Rate of more severe shigellosis	Severe or moderate diarrhoea# with fever or with one or more severe constitutional/enteric symptoms, **OR** dysentery	Dysentery defined as ≥2 Grade 3, 4, or 5 loose stools with gross blood (hemoccult positive) in 24 h *AND* [oral temperature ≥38.0 °C *OR* ≥1 severe constitutional/enteric symptom^b^]
5	Shedding of *S. sonnei* strain 53G	Positivity of at least one stool sample either by culture or quantitative polymerase chain reaction or both	
6	Severe diarrhoea	≥6 Grade 3–5 stools **OR** >800 gs of Grade 3–5 stools within 24 h **OR** required medical intervention.	Medical intervention defined as intravenous fluids administration or anticipation of antibiotic treatment before the 5th day after challenge.
7	More severe diarrhoea	≥10 Grade 3–5 stools **OR** >1000 gs of Grade 3–5 stools within 24 h or required medical intervention.	Medical intervention is defined as emergency room visit or hospitalisation for hypotensive shock.
8	Dysentery	Grade 3–5 stool with gross blood on ≥2 occasions within 24 h and presence of constitutional/enteric symptoms^c^	
9	Weight of all Grade 3–5 stools	≥8 Grade 3–5 stools or >800 gs of Grade 3–5 stools within 24 h	
10	Total number of all Grade 3–5 stools		
11	Confirmed *S. sonnei* 53 G shedding and moderate/severe diarrhoea	Confirmed *S. sonnei* 53 G shedding, **AND** moderate# or severe diarrhoea, **OR** presence of oral temperature ≥38•5 °C, **OR** presence of one or more severe intestinal symptoms, **OR** dysentery.	Dysentery defined as Grade 3, 4, or 5 stools with gross blood on ≥ 2 occasions within 24 h and presence of constitutional/enteric symptoms^c^ Intestinal symptoms included abdominal pain, cramping, nausea, vomiting, gas, anorexia
12	Disease not fulfilling the protocol primary case definition for shigellosis associated or not with mild to moderate symptoms		Associated symptoms included passing loose stool (not meeting the protocol definition of moderate or severe diarrhoea), abdominal pain, abdominal cramps, gas, anorexia, nausea, headache, myalgia, malaise, arthralgia, fever, vomiting and intravenous fluid administration.

CHIM, controlled human infection model. * Shedding of *S. sonnei* strain 53 G was defined as positivity of at least one stool sample either by culture or quantitative polymerase chain reaction or both.

Note: Constitutional/enteric symptoms were:

a nausea, abdominal pain, abdominal cramping, myalgia, arthralgia, malaise.

b headache, fatigue, arthralgia, malaise, myalgia, chills, nausea, abdominal cramping, abdominal pain, gas, anorexia, vomiting.

c headache, fatigue, arthralgia, malaise, myalgia, chills, nausea, abdominal cramping, abdominal pain, gas, anorexia, vomiting, and fever. Stools were graded as follows: Grade 1 (firm formed), Grade 2 (soft formed), Grade 3 (viscous opaque liquid or semi-liquid which assumes the shape of the bowl), Grade 4 (watery opaque liquid), Grade 5 (clear watery or mucoid liquid).

# Moderate diarrhoea was defined as [4–5 Grade 3–5 stools *OR* 400 – 800 gs of Grade 3–5 stools within 24 h] *AND* [oral temperature ≥38•0 °C *OR* ≥1 moderate constitutional/enteric symptom].

Other objectives included the evaluation of immunogenicity in terms of anti-*S. sonnei* LPS serum IgG geometric mean concentration (GMC) at each timepoint of blood sample collection and in terms of serum bactericidal activity (SBA) against *S. sonnei* prior to administration and 28 days post-administration of each 1790GAHB vaccine/placebo dose. Reactogenicity and safety were also assessed. Other exploratory objectives were part of the protocol and respective results will be disclosed in a subsequent manuscript.

### 2.3 Statistical analyses

#### 2.3.1 Sample size

Based on the percentage of participants with a seroresponse (defined as an increase in post-vaccination anti-*S. sonnei* LPS serum IgG concentrations of at least 50% for participants with pre-vaccination levels >50 enzyme linked immunosorbent assay (ELISA) units (EU), or an increase of at least 25 EU for participants with pre-vaccination levels ≤50 EU) observed in previous studies, [14, 15] a VE of 70% was assumed. Based on the results obtained by CCHMC in volunteers challenged with 1500 CFU, an attack rate (AR) for the primary case definition of 58% in the placebo group was assumed. A total number of 21 confirmed cases was needed to demonstrate that the LL of the two-sided 90% CI for the VE was above 0% with 80% power (by one proportion power analysis, one-sided test, one-sided alpha = 5%). Considering an AR of 58% in the placebo group and a percentage of non-evaluable participants of 22%, a sample size of approximately 72 individuals (36 per group / 18 per cohort) was estimated to reach the 21 shigellosis cases. The sample size was determined using the PASS 12.0.2 software.

#### 2.3.2 Analyses of efficacy

All efficacy analyses were conducted in the per-protocol set (PPS), including all participants with available data in the full analysis set who correctly received the vaccine/placebo and had no major protocol deviation.

For the primary objective, VE was evaluated at the end of study period, as 1-risk ratio (RR) where RR is the proportion of participants meeting defined case definition for shigellosis in the 1790GAHB group over the proportion in the placebo group, together with 90% CIs. Additionally, Barnard's unconditional exact test was conducted (the LL of the 90% two-sided exact unconditional CI for VE calculated with the Miettinen-Nurminen method is above 0 if the p-value of the one-sided Barnard test is below 5%). Additional details on secondary efficacy endpoints are provided in appendix p 1.

#### 2.3.3 Analysis of safety

Solicited adverse events (AEs) and unsolicited AEs were assessed in the corresponding safety sets that included all vaccinated participants with available solicited/unsolicited safety data. More details are provided in appendix pp 3–4.

#### 2.3.4 Analysis of immunogenicity

Immunogenicity analyses were conducted in the PPS. Results were presented overall and separately for participants who did or did not develop shigellosis (primary case definition) during the 8-day inpatient period. A modified ELISA was used in this study (appendix p 2) as compared to previous studies [14], [15], [16]. The antibody response cut-off of 268 ELISA units (EU)/mL used in this assay corresponds to the formerly used threshold of 121 EU [14] which was estimated to correspond to the median endpoint titre of 1:800 reported in the sera of convalescent individuals previously infected with *S. sonnei* [11].

Anti-*S. sonnei* LPS serum IgG was measured at D1, 7, and 28 days post-each 1790GAHB/placebo dose and post-challenge. For each group, GMCs and their 95% CIs were computed by exponentiating (base 10) the mean and 95% CIs of the log10 ELISA concentrations. Median anti-*S. sonnei* LPS IgG concentrations were also calculated. The number and percentage of participants with anti-*S. sonnei* LPS IgG ≥268 EU/mL with related 95% CIs were also calculated. Bactericidal activity was assessed by a luminescent serum bactericidal assay (L-SBA) [19] at D1 and 28 days post-each vaccination. SBA geometric mean titres (GMTs) were tabulated with their 95% CIs. Antibody concentrations below the lower limit of quantification (LLOQ) (22 EU/mL for ELISA and inhibition concentration [IC]50 of 100 for L-SBA) were set to half that limit for the purpose of analysis. Within-subject geometric mean ratios were computed for GMC/GMT at each post-vaccination time points versus baseline levels and post-challenge versus pre-challenge by exponentiating the mean within-subject differences in log-transformed titres and the corresponding CIs.

All statistical analyses were carried out using Statistical Analysis Systems 9.4.

#### 2.3.5 Role of the funding source

This study was funded by the Bill and Melinda Gates Foundation and GlaxoSmithKline Biologicals SA. GlaxoSmithKline Biologicals SA also took responsibility for all costs associated with the preparation of clinical documents and the development and publishing of the present manuscript.

## 3 Results

### 3.1 Demographics

**Fig. 1:**
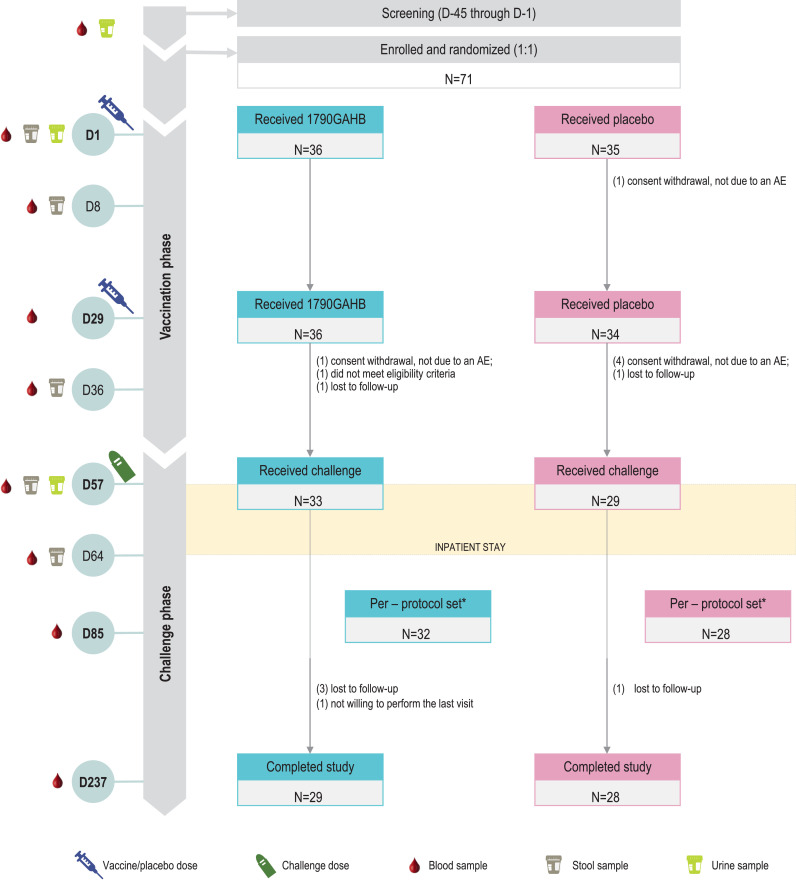
Study design and participant flowchart N, number of participants; D, day; AE, adverse event. * one participant in each group did not complete the inpatient stay and was excluded from the per-protocol set. Both participants completed the study.

A total of 71 adults were enroled and vaccinated: 36 participants in the 1790GAHB group and 35 in the Placebo group; 33 and 29 participants, respectively, received the challenge dose (1190 CFU [Cohort 1], 1480 CFU [Cohort 2], 1520 CFU [Cohort 3], and 1050 CFU [Cohort 4]). One participant in Cohort 2 received a challenge dose of 1350 CFU, 2 participants in Cohort 4 received 1130 CFU. Twenty-nine 1790GAHB and 28 placebo recipients completed the study (Fig. 1). The most common reasons for not completing the study were “lost to follow-up” in the 1790GAHB group and “consent withdrawal not due to an AE” in the Placebo group. Demographic characteristics were balanced between groups (Table 2).

**Table 2:** Demographic characteristics of study participants.

	1790GAHB group		Placebo group		Total	
	Exposed set (*N* = 36)	Per-protocol set (*N* = 32)	Exposed set (*N* = 35)	Per-protocol set (*N* = 28)	Exposed set (*N* = 71)	Per-protocol set (*N* = 60)
Age (mean±SD), years	36·2 ± 9·9	36·4 ± 10·0	35·2 ± 8·5	34·8 ± 9·1	35·7 ± 9·2	35·7 ± 9·6
Male, n (%)	22 (61·1)	20 (62·5)	18 (51·4)	16 (57·1)	40 (56·3)	36 (60·0)
Female, n (%)	14 (38·9)	12 (37·5)	17 (48·6)	12 (42·9)	31 (43·7)	24 (40·0)
Ethnicity, n (%)						
Not Hispanic or Latino	35 (97·2)	31 (96·9)	34 (97·1)	27 (96·4)	69 (97·2)	58 (96·7)
Hispanic or Latino	1 (2·8)	1 (3·1)	1 (2·9)	1 (3·6)	2 (2·8)	2 (3·3)
Race, n (%)						
Black or African American	24 (66·7)	20 (62·5)	28 (80·0)	24 (85·7)	52 (73·2)	44 (73·3)
White	10 (27·8)	10 (31·3)	6 (17·1)	4 (14·3)	16 (22·5)	14 (23·3)
Other	2 (5·6)	2 (6·3)	1 (2·9)	0 (0·0)	3 (4·2)	2 (3·3)

N, total number of participants; n (%), number (percentage) of participants in each category; SD, standard deviation·

### 3.2 Vaccine efficacy

During the 8-day post-challenge period, 15 participants in the 1790GAHB group and 12 in the Placebo group developed shigellosis according to the primary case definition, resulting in an AR of 46.9% (1790GAHB group) and 42.9% (Placebo group). VE was −9.4% (90% CI: −96.7–33.7; *P*=value 0.4266) (Table 3). As the LL of the 90% CI for VE is below 0, no efficacy was demonstrated according to the primary endpoint. Efficacy was not demonstrated for any other secondary endpoints, including lack of significant difference in quantitative shedding (measured by qPCR as CFU/g per day) between vaccinated and placebo groups (Wilcoxon rank test *P* = 0·6858). The primary endpoint was also evaluated in the full analysis set, leading to a VE of −9.8% (90% CI: −97.8–33.7, P-value=0·3944).

**Table 3:** Vaccine efficacy of the 1790GAHB vaccine versus placebo against evaluated endpoints (per-protocol set).

	Endpoint	Measured outcome					Vaccine efficacy	p-value*
		1790GAHB group (*N* = 32)		Placebo group (*N* = 28)				
		n	Value (90% CI)	n	Value (90% CI)		% (90% CI)	
1	Rate of shigellosis, 1st definition (primary endpoint) (%)	15	46·9 (31·5; 62·7)	12	42·9 (26·9; 60·0)		−9·4 (−96·7; 33·7)	0·4266
2	Rate of shigellosis, 2nd definition (%)	15	46·9 (31·5; 62·7)	9	32·1 (17·9; 49·4)		−45·8 (−173·1; 17·4)	0·1485
3	Rate of shigellosis, 3rd definition (%)	15	46·9 (31·5; 62·7)	10	35·7 (20·8; 53·0)		−31·3 (−130·1; 22·9)	0·2917
4	Rate of more severe shigellosis (%)	5	15·6 (6·4; 30·1)	4	14·3 (5·0; 29·8)		−9·4 (−275·6; 62·7)	0·4787
5	Shedding of *S. sonnei* strain 53 G (%)	31	96·9 (86·0; 99·8)	28	100·0 (89·9; 100·0)		3·1 (−7·0; 14·0)	0·2763
6	Severe diarrhoea (%)	13	40·6 (26·0; 56·7)	7	25·0 (12·4; 41·9)		−62·5 (−270·3; 17·0)	0·1228
7	More severe diarrhoea (%)	11	34·4 (20·6; 50·4)	5	17·9 (7·3; 33·9)		−92·5 (−534·8; 11·7)	0·0965
8	Dysentery (%)	11	34·4 (20·6; 50·4)	8	28·6 (15·1; 45·7)		−20·3 (−137·4; 37·2)	0·3479
9	Mean weight of all Grade 3–5 stools (grams)		114·2 (41·2; 316·2)		147·3 (58·7; 369·4)			0·7579
10	Total number of all Grade 3–5 stools⁎⁎	327		172				0·1362⁎⁎⁎
11	Confirmed *S. sonnei* 53 G shedding and moderate/severe diarrhoea (%)	15	46·9 (31·5; 62·7)	12	42·9 (26·9; 60·0)		−9·4 (−96·7; 33·7)	0·4266
12	Disease not fulfilling the protocol primary case definition for shigellosis associated or not with mild to moderate symptoms (%)	16	50·0 (34·4; 65·6)	17	60·7 (43·5; 76·2)		17·6 (−23·4; 47·3)	0·2917

⁎ P-value from one-sided test, not adjusted for multiple comparisons, with #1 being the primary endpoint and the remaining 11, secondary endpoints.

⁎⁎ Mean number of stools per participant (± standard deviation): 28.0 ± 13.0 (1790GAHB group) and 13.9 ± 6.6 (Placebo group).

⁎⁎⁎ p-value calculated to compare the mean number of stools. N, number of participants; n, number of participants in each category; CI, confidence interval. Note: P-values for endpoints 2–8 and 11–12 were calculated in post-hoc analyses.

### 3.3 Safety

**Fig. 2:**
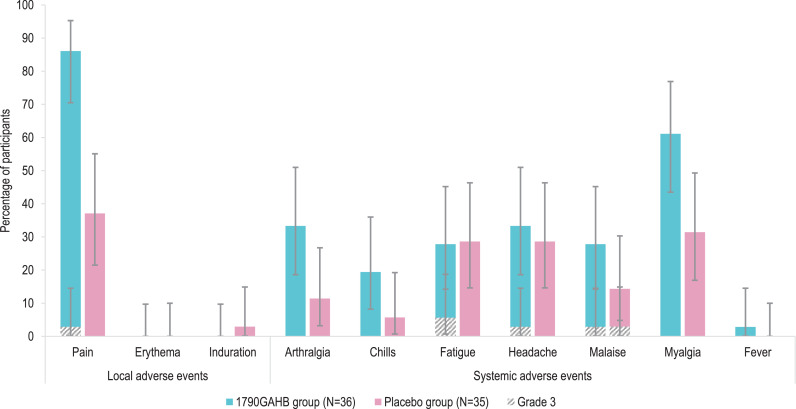

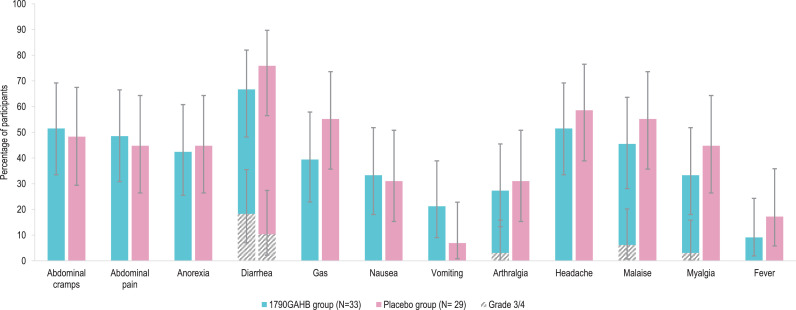
Solicited adverse events 7 days post-vaccination (2 doses) (A) and 8 days post-challenge with *S. sonnei* (B) (solicited safety set) N, number of participants in each group. Note: Error bars depict 95% confidence intervals. Definitions of solicited adverse event grades are detailed in Table S1 (appendix pp 3-4).

During the 7-day post-vaccination period, 31 (86.1%) participants in the 1790GAHB group and 13 (37.1%) participants in the Placebo group reported at least one solicited local AE and 24 (66.7%) and 17 (48.6%) participants, respectively, reported at least one solicited systemic AE. All solicited local AEs were mild to moderate in severity (grading of AEs detailed in appendix pp 3–4, **Table S1**) except for one case of Grade 3 pain in the 1790GAHB group (Fig. 2**A**). The most frequent systemic AE was myalgia in both groups. All reported solicited systemic AEs were mild to moderate in severity except for four Grade 3 AEs in the 1790GAHB group after the second dose (fatigue [2 participants], headache [1 participant], and malaise [1 participant]) and one Grade 3 AE (malaise) in the Placebo group after the first dose. One Grade 1 fever event (≥38.0 °C–<39.0 °C) was observed in the 1790GAHB group after the first dose.

During the 8-day post-challenge period, the most frequently reported solicited event was diarrhoea in 22 (66.7%) participants in the 1790GAHB group and 22 (75.9%) in Placebo group. Grade 4 diarrhoea was reported by 6 (18.2%) vaccinees and 3 (10.3%) placebo recipients (Fig. 2**B**).

During the 28-day post-vaccination period, 18 (50.0%) participants in the 1790GAHB group and 13 (38.2%) participants in the Placebo group reported at least one unsolicited AE (appendix p 5, **Table S2**); 5 (13.9%) and 3 (8.8%) participants, respectively, experienced an event considered by the investigator as causally related to vaccine/placebo administration. During the 28-day post-challenge period, at least one unsolicited AE was reported by 20 (60.6%) participants in the 1790GAHB group and 17 (58.6%) participants in the Placebo group (appendix p 6, **Table S3**); 12 (36.4%) and 12 (41.4%) participants, respectively, experienced an event considered causally related to challenge dose. No severe unsolicited AE was reported during the 28-day post-vaccination/challenge periods. No deaths, nor AE of special interest (symptomatic neutropenia) were reported during the study. However, eight events of Grade 1 neutropenia (i.e., absolute neutrophil count [ANC] 1800–1500 cells/µL) were reported in 2 subjects in the 1790GAHB group; nine events of Grade 1 neutropenia and three events of Grade 2 neutropenia (i.e., ANC 1499–1000 cells/µL) were reported in 5 subjects in the Placebo group. All events were transient and clinically asymptomatic. The neutrophil values returned above the threshold values set in the study protocol, with a duration between 1 and 21 days after detection.

No serious AE (SAE) was reported in the 1790GAHB group. In the Placebo group, one participant experienced four SAEs following the challenge dose: deep vein thrombosis (moderate intensity) and pelvic venous thrombosis (severe) at day 107, haematoma (severe) at day 121, and carotid artery aneurysm at day 130 from challenge. All events were assessed by the investigator as unrelated to any study treatment and were resolved.

There were no AEs that led to subject premature withdrawal from the study, any dose reduction, interruption, or delay in study vaccination.

### 3.4 Immunogenicity

**Fig. 3:**
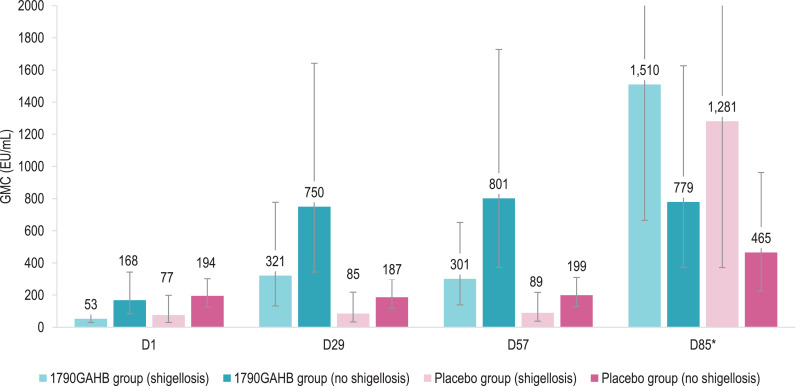

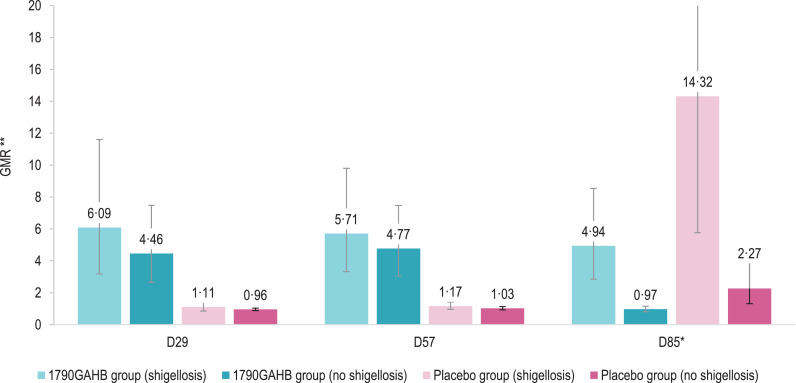
Anti-*S. sonnei* LPS serum IgG geometric mean concentrations (A) and geometric mean ratios (B) in participants who did or did not develop shigellosis post-challenge (per-protocol set) LPS, lipopolysaccharide; IgG, immunoglobulin G; 1790GAHB / Placebo group (shigellosis), participants who received the 1790GAHB vaccine / placebo and developed shigellosis after the bacterial challenge; 1790GAHB / Placebo group (no shigellosis), participants who received the 1790GAHB vaccine / placebo and did not develop shigellosis after the bacterial challenge; GMC, geometric mean concentration; EU, enzyme-linked immunosorbent assay units; D1, baseline; D29, 28 days post-first 1790GAHB/placebo vaccination; D57, 28 days post-second 1790GAHB/placebo vaccination; D85, 28 days post-challenge dose; GMR, geometric mean ratio. Note: Error bars depict 95% confidence intervals. *The upper limits of the 95% confidence intervals at D85 are 3430•58 (1790GAHB group [shigellosis]) and 4419•02 (Placebo group [shigellosis]) in panel A and 35•56 (Placebo group [shigellosis]) in panel B. ^⁎⁎^ GMRs are calculated relative to D1 for D29 and D57, and relative to D57 for D85.

Baseline anti-*S. sonnei* LPS serum IgG GMCs were 97.6 EU/mL in the 1790GAHB group and 130.5 EU/mL in the Placebo group (Table 4). In both groups, baseline antibody levels were higher amongst participants who did not develop versus those who developed shigellosis post-challenge: 168.1 versus 52.8 EU/mL (*P* = 0·0133 from *t*-test on log_10_ transformed data) in 1790GAHB and 194.5 versus 76.6 EU/mL (*P* = 0·0690) in placebo recipients (Fig. 3**,** appendix p 7, **Table S4**). At 28 days post-first vaccination (D29), in the 1790GAHB group, GMC increased to 749.8 EU/mL in participants who did not develop shigellosis post-challenge and 321.2 EU/mL in participants who developed shigellosis post-challenge. No substantial increase in antibody levels was shown at D57, after the second vaccination. No increases in antibody GMCs were observed after any dose in the Placebo group, but, as for the 1790GAHB group, the pre-challenge median anti-*S. sonnei* LPS serum IgG concentration was higher. Amongst participants who did not develop shigellosis compared to participants who developed the disease following the challenge dose (*P* = 0·1050 [Wilcoxon test] in the 1790GAHB group and *P* = 0·0373 in the Placebo group) (appendix p 11, **Figure S2**).

**Table 4:** Percentage of participants with anti-*S. sonnei* LPS serum IgG ≥268 EU/mL, geometric mean concentrations and geometric mean ratios by time point (per-protocol set).

	% ≥268 EU/mL (95% CI)			GMC [EU/mL] (95% CI)				GMR (95% CI)	
	1790GAHB group (*N* = 32)	Placebo group (*N* = 28)		1790GAHB group (*N* = 32)	Placebo group (*N* = 28)		Ratio	1790GAHB group (*N* = 32)	Placebo group (*N* = 28)
D1	31 (16; 50)	21 (8; 41)		97·63 (59·87; 159·21)	130·47 (81·06; 209·98)			–	–
D8	47 (29; 65)	18 (6; 37)		227·24 (130·55; 395·55)	136·83 (89·21; 209·87)		D8/D1	2·33 (1·65; 3·28)	1·05 (0·95; 1·16)
D29	69 (50; 84)	21 (8; 41)		503·89 (283·91; 894·29)	133·09 (82·89; 213·71)		D29/D1	5·16 (3·50; 7·61)	1·02 (0·91; 1·14)
D36	66 (47; 81)	18 (6; 37)		558·81 (324·54; 962·18)	132·62 (84·17; 208·95)		D36/D1	5·72 (3·99; 8·21)	1·02 (0·93; 1·11)
D57	69 (50; 84)	21 (8; 41)		506·48 (293·97; 872·60)	141·43 (90·39; 221·29)		D57/D1	5·19 (3·73; 7·21)	1·08 (0·98; 1·19)
D64	69 (50; 84)	39 (22; 59)		560·95 (317·73; 990·36)	196·53 (127·59; 302·71)		D64/D57	1·11 (0·94; 1·31)	1·39 (1·09; 1·77)
D85*	84 (66; 95)	65 (44; 83)		1050·25 (618·67; 1782·92)	742·37 (379·46; 1452·38)		D85/D57	2·03 (1·37; 2·99)	5·32 (2·91; 9·73)

LPS, lipopolysaccharide; IgG, immunoglobulin G; EU, enzyme-linked immunosorbent assay units; N, number of participants; GMC, geometric mean concentration; GMR, within-subjects geometric mean ratio; D, day; CI; confidence interval. *N = 31 in the 1790GAHB group and *N* = 26 in the Placebo group.

**Fig. 4:**
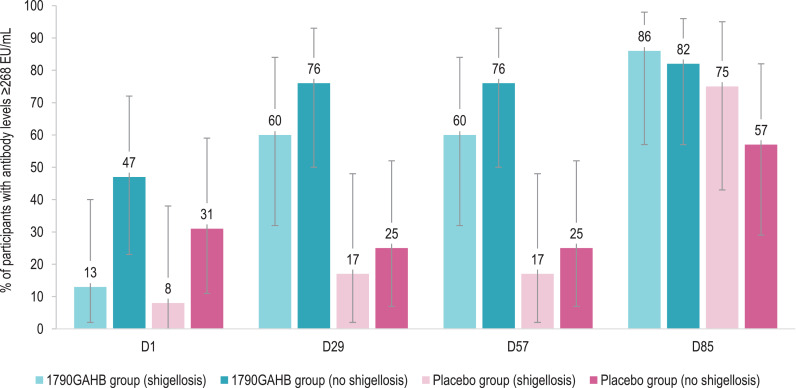
Percentage of participants with anti-*S. sonnei* LPS serum IgG antibody levels ≥268 EU/mL in participants who did or did not develop shigellosis post-challenge (per-protocol set) LPS, lipopolysaccharide; IgG, immunoglobulin G; EU, enzyme-linked immunosorbent assay units; 1790GAHB / Placebo group (shigellosis), participants who received the 1790GAHB vaccine / placebo and developed shigellosis after the bacterial challenge; 1790GAHB / Placebo group (no shigellosis), participants who received the 1790GAHB vaccine / placebo and did not develop shigellosis after the bacterial challenge; D1, baseline; D29, 28 days post-first 1790GAHB/placebo vaccination; D57, 28 days post-second 1790GAHB/placebo vaccination; D85, 28 days post-challenge dose. Note: Error bars depict 95% confidence intervals.

By D57, 69% of vaccinees and 21% of placebo recipients achieved antibody concentration ≥268 EU/mL (Table 4). Amongst participants who did not develop shigellosis, 47% of 1790GAHB and 31% of placebo recipients had antibody concentrations ≥268 EU/mL at baseline and 76% (1790GAHB group) and 25% (Placebo group) at both D29 and D57 (Fig. 4**,** appendix p 7, **Table S4**). By D85 (28 days post-challenge), percentages increased to 82% (1790GAHB group) and 57% (Placebo group). Amongst participants subsequently developing shigellosis, 13% (1790GAHB group) and 8% (Placebo group) had anti-*S. sonnei* LPS serum IgG ≥268 EU/mL at baseline; these percentages increased to 60% and 17% by D29, remained unchanged at D57, then further increased to 86% and 75% by D85 (Fig. 4**,** appendix p 7, **Table S4**).

The anti-*S. sonnei* LPS serum IgG levels induced by the challenge agent, evaluated at 28 days post-challenge dose (D85), was different in both treatment groups between participants who did not and did develop shigellosis. Amongst participants who did not develop shigellosis, there was a post-challenge 2.3-fold increase in the placebo group, while 1790GAHB recipients did not show any post-challenge increase. amongst participants who developed shigellosis, GMCs increased 4.9-fold in the 1790GAHB group and 14.3-fold in the Placebo group, compared to pre-challenge values (Fig. 3**,** appendix p 7, **Table S4**).

As shown for ELISA, peak bactericidal antibody titres, tested by l-SBA, were achieved after the first vaccine dose (D29) and SBA GMTs in the vaccine group were statistically higher in participants who did not develop shigellosis post-challenge compared to participants who developed the disease, both at baseline (*P* = 0·0062 from *t*-test) and at D29 (*P* = 0·0004 from *t*-test) (appendix p 8 and p 12, **Table S5, Figure S3**). Comparability amongst groups with low bactericidal antibody (i.e., GMT less than 100), such as placebo recipients or vaccinees who developed shigellosis after challenge, was affected by the limited sensitivity of the assay (LLOQ=100).

## 4 Discussion

This study intended to primarily assess the efficacy of 1790GAHB vaccine to prevent shigellosis following a challenge dose of *S. sonnei* 53 G in healthy *Shigella*-naïve adults from the United States. The proportion of cases of shigellosis defined according to the primary endpoint was similar between vaccine and placebo recipients, indicating a lack of efficacy for the 1790GAHB vaccine in this trial. Although not statistically significant, there was an apparent trend for increased occurrence of some more severe endpoints in the vaccinated group. The imbalance between the vaccine and placebo recipients in the anti-LPS antibody levels at baseline might be one of the possible explanations for this observation. Future studies are planned to evaluate anti-protein responses and the quality of antibody response and will help shedding more light on this aspect.

Safety results confirmed the acceptable safety profile of 1790GAHB demonstrated in previous clinical trials [14], [15], [16]. Solicited and unsolicited AEs were more frequent in 1790GAHB versus Placebo group post-vaccination. No vaccination-related SAE, symptomatic neutropenia, or deaths were reported. A total of 20 neutropenia events were identified, all transient and clinically asymptomatic. Most of those observed events occurred in the Placebo group and in participants of Black or African-American ethnicity (five out of seven participants) who are known to generally have lower levels of neutrophils [20]. These data support previous observations that targeted clinical laboratory reference intervals should be used based on the specific study population [15].

Overall, pre-challenge median anti-*S. sonnei* LPS IgG antibody levels were consistently higher in volunteers who did not develop shigellosis than in those who developed the disease. This is in agreement with evidence from previous studies and the belief of the scientific community that anti-LPS IgG responses play a significant role in protection against shigellosis [11, 21, 22]. A study assessing the association between anti-*S. sonnei* LPS serum IgG and natural immunity to shigellosis in adults reported that the risk of developing symptomatic *S. sonnei* infection was estimated to be 5.5-fold higher for participants with low pre-exposure IgG antibody levels compared to those with higher values (threshold: 1:200) [23]. Of note, some placebo recipients with low anti-LPS antibody concentrations did not develop shigellosis in this trial, which suggests that other factors may contribute to protection. Assessment of the role of other immunological readouts in clinical protection are planned and will be addressed in a future publication. This will include evaluation of anti-LPS serum IgA, IgM, or IgG subclass responses and of antibody avidity, and of anti-proteins (including non-vaccine antigens) responses which could help to elucidate the differences in priming between natural exposure and vaccination and possibly explain the apparent trend for an increased incidence of various clinical markers of shigellosis (including those for more severe shigellosis) post-challenge, in vaccinated individuals. Currently, SBA analysis has been performed on sera depleted of anti-*S. sonnei* LPS antibodies. In line with recent preclinical data [24], no bactericidal activity was detected, suggesting that anti-protein antibodies may not have a major role in protection or at least that their role is not mediated by bactericidal activity.

Based on the differences in the magnitude of pre-challenge antibody concentrations between vaccinees who did or did not develop shigellosis, insufficient immunogenicity of the vaccine can be considered as the main root cause for the absence of efficacy. The insufficient antibody response and associated lack of protection in many vaccinees might be related to the relatively low OAg dose. 1790GAHB contained 1.5 µg of *S. sonnei* OAg, much less than the 25 µg used in a previous conjugate vaccine demonstrating 74% protective efficacy against shigellosis in adults [25], or the 10 µg used in the bioconjugate vaccine Flexyn2a [26] and the synthetic carbohydrate-based vaccine SF2a-TT15 [27] against *S. flexneri* 2a. For the former, a 48-fold increase in anti-*S. sonnei* LPS serum IgG was observed two weeks after a first vaccine dose [28], compared to a 5.16-fold increase in antibody GMC observed at four weeks post-vaccination with 1790GAHB in the current study. Similarly, the SF2a-TT15 formulation containing 10 µg of SF2a OAg induced a much stronger immune response than that containing 2 µg [27], supporting the rationale that an OAg content much higher than 1.5 µg is needed for the GMMA vaccine as well. Additionally, evidence of immunogenicity dose response from early 1790GAHB trials, particularly in naïve subjects with undetectable antibody at baseline [29], implies that a vaccine containing a higher amount of *S. sonnei* OAg may indeed be more immunogenic and ultimately efficacious. The 1.5 µg of OAg in 1790GAHB was kept mainly for two reasons: 1) this dose was able to induce antibody concentrations higher than the median antibody concentration in convalescents after natural exposure [29] and 2) for safety consideration, it was preferred not to increase the amount of GMMA-associated proteins and lipid A, also in light of the need to ultimately have a multicomponent GMMA *Shigella* vaccine formulation for broad coverage [30]. A new *S. sonnei* GMMA construct, currently under evaluation at GVGH, expresses increased OAg density resulting in a higher OAg to protein and lipid A ratio. The use of this GMMA construct may allow a higher OAg dose without the risk of potentially affecting the safety profile of the vaccine.

Another cause for the reduced immunogenicity might be the suboptimal immunization schedule. We previously demonstrated that a booster dose given approximately three years post-primary vaccination elicited high anamnestic responses even in adults primed with very small amount of 1790GAHB (0.06/1 µg of OAg/protein) [16]. In the current study, the second 1790GAHB dose did not increase antibody levels, suggesting that the one-month interval between the two doses may have been too short to be able to increase immune responses in 1790GAHB recipients. This is in line with results obtained for other investigational vaccines with similar vaccination schedules [9,26,31,32]. Similarly, functional antibodies were induced by the first 1790GAHB dose but no significant increase was observed following the administration of the second dose. A more widely spaced immunization schedule for a GMMA-based vaccine has not been evaluated but might induce higher antibody levels and conceivably greater protection against shigellosis.

Although clinical efficacy was not demonstrated, the evaluation of antibody responses induced after the challenge administration was useful to identify some differences between vaccine and placebo recipients. Interestingly, as shown in Fig. 4, participants who did not develop shigellosis in the 1790GAHB group were the only study participants who did not show any anti-*S. sonnei* LPS antibody increase following challenge administration. This may suggest that, in this group, vaccination conferred protection against bacterial multiplication, the cause of clinical shigellosis above a certain threshold.

Human challenge trials have been repeatedly used to elucidate disease pathogenesis and human responses to infection, including identification or confirmation of possible correlates of protection, but also in the clinical development of vaccines. Particularly in cases where field efficacy trials are virtually impossible, CHIM trials have provided efficacy data for registration, as shown by the recent approval of a cholera vaccine for travellers [33]. However, there has been considerable discussion on the appropriateness of CHIM trials established in high-income countries to evaluate VE and consequently on the potential value of conducting CHIM trials in LMIC settings, where *Shigella* is endemic [33]. The limited results available suggest that the variability of the pre-existing *Shigella*-specific antibody levels in LMIC populations may complicate ensuring an adequate AR and alter the reproducibility of the trial [34]. Other discussions have been centred on the reliability and validity of potential serological correlates of protection and measures of immunogenicity, established in field trials, and transposed under the artificial CHIM conditions (which include higher doses of the challenge agent – administered following neutralization of gastric acidity – as compared with natural infection) [33]. Indeed, our trial showed that 39% of the participants with pre-challenge antibody concentration ≥268 EU/mL (i.e., Cohen's median antibody concentration in convalescents [11]) developed shigellosis and even amongst those with a pre-challenge concentration above 536 EU/mL (i.e., twice as high as the Cohen's threshold after natural exposure), 31% still developed shigellosis (data not shown). These data not only suggest that other immune mechanisms are also important and may serve as mechanistic correlates of protection against disease (e.g., IgA), but also reinforce the concern raised about the artificiality of this model that may prematurely halt development of a vaccine potentially efficacious in field settings [35]. Field studies assessing clinical efficacy in a target paediatric population of endemic regions might address these concerns and produce more conclusive results, particularly when the epidemiology of the disease would allow this with approachable trial sizes.

An additional limitation of this trial is related to the screening threshold adopted in the challenge model (i.e., a titre of 1:2500, corresponding to 1940 EU/mL as tested with the GVGH ELISA), that allowed enrolment of a large proportion of participants with a specific baseline antibody level close to or above levels considered protective in field conditions [23]. Of the 60 participants included in the immunogenicity analysis of the current study, 31% had baseline antibody concentration ≥268 EU/mL in the 1790GAHB group and 21% in the Placebo group. Logistic regression model applied to the entire study population showed a reduction of odds ratio (OR) of shigellosis in participants with higher antibody at baseline (data not shown). The p-value for the OR was significant for both ELISA (*P* = 0.003) and SBA (*P* = 0.0491), demonstrating a strong association between antibody at baseline and risk of disease and confirming that several subjects were already protected at baseline. Moreover, the rapid increase of antibody level at D8 as well as the lack of increase after the second dose in the 1790GAHB group may suggest a post-first dose secondary response. This likely explains why the AR in the Placebo group was only 43% instead of the planned 58%. The enrolment of a high number of individuals with pre-existing elevated antibody levels has implications on studies intrinsically characterized by limited power, including the interpretation of the results. Therefore, in future *Shigella* CHIM trials, the use of a more conservative screening threshold should be considered, although this might be perceived as increasing the artificiality of the model. As the purpose of the screening is to select a Shigella-naïve population – to mirror as much as possible the naïve immunological profile of the infant target population and, ultimately, to reduce bias of interpretation and increase the power of the study – we believe there is a solid rationale for the use of a different threshold.

In summary, the 1790GAHB candidate vaccine showed an acceptable safety profile and induced anti-*S. sonnei* LPS serum IgG but did not show clinical protection against shigellosis in this *S. sonnei* CHIM trial. Antibody levels, considered to be protective in field conditions (i.e., ≥268 EU/mL), were achieved by 69% of vaccinees, but were insufficient to fully protect them from the *S. sonnei* challenge. To strengthen the anti-*S. sonnei* LPS response to vaccination and its protective potential, the *S. sonnei* GMMA OAg dose in the four-component GMMA *Shigella* vaccine, currently under development, should be significantly increased, the administration schedule more widely spaced, and its immunogenicity tested before progression to further trials in the target populations.

## 5 Authors contribution

All authors attest they meet the ICMJE criteria for authorship. RWF, VC, PF, AGWN, JPG, GLC, IDR, JA, EM, RWK, AS, LBM, and AP were involved in the conception or the design of the study. RWF, SP, MMM, MD, FN, AES, OR, AA, LC, KAC, and FM participated in the collection or generation of the study data, PF and RWK also contributed to the study with materials/analysis tools. RWF, VC, PF, SP, MMM, MD, FN, OR, and AA performed the study. RWF, VC, PF, JPG, GLC, FN, AES, OR, AA, LC, KAC, RWK, FM, RR, AS, RR, LBM, and AP were involved in the analyses and interpretation of the data. All authors revised the manuscript critically for important intellectual content and gave final approval to submit for publication.

## Declaration of Competing Interest

AP, LBM, RR, GLC, PF, IDR, FM, FN, EM, JA, AA, OR and VC are employees of the GSK group of companies. AP, LBM, EM, and JA hold shares in the GSK group of companies. JA, LBM, AS, GLC, AGWN, PF, and IDR report grants from the Bill and Melinda Gates Foundation and the Wellcome Trust during the conduct of the study. LBM reports grants from the Bill and Melinda Gates Foundation and the Wellcome Trust, outside the submitted work. AS, AGWN, and JPG were employees of the GSK group of companies at the time of study conduct. AA report personal fees from GSK Vaccines Institute for Global Health during the conduct of the study. AS hold shares in the GSK group of companies. LBM and AS are inventors of patents owned by the GSK group of companies and relevant to *Shigella* vaccine. AES, KAC, LC, MMcN, and RWK report grants from the GSK group of companies during the conduct of the trial. MMcN reports grants from NIH, outside the submitted work. MD, SP, and RWF have nothing to disclose.

## Appendix Supplementary materials

Image, application 1
