# Supplementary material for: Efficacy, safety, and immunogenicity of the Shigella sonnei 1790GAHB GMMA candidate vaccine: Results from a phase 2b randomized, placebo-controlled challenge study in adults
Source: eClinicalMedicine. 2021 Aug 13;39:101076. doi: 10.1016/j.eclinm.2021.101076 (PMC8367798; doi:10.1016/j.eclinm.2021.101076)
Supplement: Supplementary file 1 [file mmc1.docx]

Appendix

Efficacy, safety, and immunogenicity of the *Shigella sonnei* 1790GAHB GMMA candidate vaccine: Results from a phase 2b randomized, placebo-controlled challenge study in adults

**Authorship:** Robert W. Frenck, Jr, MD^1^, Valentino Conti, MSc^2^, Pietro Ferruzzi, PhD^2^, Augustin G. W. Ndiaye, MD^2,#^, Susan Parker, RN^1^, Monica Malone McNeal, MS^1,3^, Michelle Dickey, MS^1,3^, Juan Paolo Granada, MD^2,4,$^, Giulia Luna Cilio, MSc^4^, Iris De Ryck, MD^4^, Francesca Necchi, PhD^2^, Akamol E. Suvarnapunya, PhD^5^, Omar Rossi, PhD^2^, Alessandra Acquaviva, MSc^2^, Lakshmi Chandrasekaran, PhD^5^, Kristen A. Clarkson, PhD^5^, Joachim Auerbach, PhD^2^, Elisa Marchetti, PhD^2^, Robert W. Kaminski, PhD^5^, Francesca Micoli, PhD^2^, Rino Rappuoli, PhD^2, 4^, Allan Saul, PhD^2 &^, Laura B. Martin, PhD^2^, and Audino Podda, MD^2^*

**Affiliation:**

^1^Division of Infectious Diseases, Cincinnati Children’s Hospital Medical Center, Cincinnati, Ohio, United States

^2^GSK Vaccines Institute for Global Health, Siena, Italy

^3^Department of Pediatrics, University of Cincinnati College of Medicine, Cincinnati, Ohio, United States

^4^GSK, Siena, Italy

^5^Department of Diarrheal Disease Research, Bacterial Diseases Branch, Walter Reed Army Institute of Research, Silver Spring, Maryland, United States

*Corresponding author

^#^Current affiliation: Takeda Pharmaceutical International AG, Zurich, Switzerland

^$^Current affiliation: Ferring Pharmaceuticals, Copenhagen, Denmark

^&^Current affiliation: Burnet Institute, Melbourne, Australia

**Corresponding author:**

Audino Podda

GSK Vaccines Institute for Global Health, Via Fiorentina 1, 53100 Siena, Italy

Email: [audino.p.podda@gsk.com](mailto:audino.p.podda@gsk.com)

Phone: +39 335 7026950

**Study participants**

Adults who provided informed consent were assessed for eligibility in the study at the end of a screening period (45 days before the first vaccine/placebo administration), during which they were screened for general health status and their medical history was recorded. Individuals were included in the study if they were 18–50 years of age, provided informed consent, were generally healthy, and negative for HIV, hepatitis B and hepatitis C. Individuals who had a previous laboratory confirmed case of disease caused by *S. sonnei* infection or an anti-*S. sonnei* LPS serum IgG titre >1:2500 at screening, corresponding to 1940 EU/mL as measured by the GVGH enzyme linked immunosorbent assay (ELISA), were excluded. Only participants who met all inclusion criteria and none of the exclusion criteria were eligible for enrolment.

**Administration of antibiotic**

Participants may be treated with antibiotics before the 5th day after challenge if:

- They have diarrhoea (any severity stool Grade 3 or higher) and two or more of the following symptoms: severe abdominal pain/cramps, severe nausea, severe headache, severe myalgia, severe arthralgia, gross blood in ≥2 stools, any fever (≥38.0ºC measured orally), or any vomiting.

- They experience unexpectedly severe events such as hypotension (disproportionate to volume loss), renal dysfunction, or altered mental state (e.g. somnolence) at the discretion of the investigators.

- A study physician determines early treatment is warranted for other reasons.

**Analysis of efficacy**

For secondary efficacy endpoints measured as proportion, vaccine efficacy and 90% confidence intervals (CIs) were calculated as outlined for the primary objective. For count endpoints (i.e. total number of Grade 3–5 stools), the risk ratio was estimated as the rate of Grade 3–5 stools in participants in the 1790GAHB group divided by the rate of Grade 3–5 stools in the Placebo group; 90% CI for this rate ratio were calculated from an unadjusted negative binomial model, to take into account possible over-dispersion of data, and the unadjusted p-value from the associated 2-tailed Wald was computed. Continuous endpoints (i.e. weight of Grade 3–5 stools per subjects, weight of Grade 3–5 stools per subject in those with at least a Grade 3–5 stools, and weight of Grade 3–5 stools accumulated from challenge to discharge per participant) were compared using unequal variance two-sample t-test in case of normality of data, unequal variance 2-sample t-test on the log values in cases where log-values were normally distributed, otherwise by Wilcoxon 2-sample test; unadjusted p-values from two-tailed test were computed. In cases of comparisons using log-values, the geometric mean as well as the arithmetic mean were calculated, with associated 90% CI.

Participants with missing values of efficacy endpoints were excluded from analyses as they were considered missing completely at random, i.e. not informative and with no impact on inferences.

**Analysis of safety**

Participants were monitored for safety throughout the study. A GSK Safety Review Team analysed blinded data to detect potential safety signals on a continuous basis. At predefined timepoints, an Independent Data Monitoring Committee (IDMC) reviewed unblinded safety data and made recommendations concerning continuation, termination, or other modifications of the study. Based on prior experience in 1790GAHB-GMMA studies,^14,15^ the occurrence of ≥ Grade 3 symptomatic neutropenia in ≥ 1 participant triggered a halting rule for enrolment of additional participants until the event was reviewed by IDMC. Grading was defined as absolute neutrophil count (ANC) 1800–1500 cells/µL (Grade 1), 1499–1000 cells/µL (Grade 2), 999–500 cells/µL (Grade 3), or <500 cells/µL (Grade 4).

Participants were observed for 30 minutes after each vaccination for occurrence of any immediate adverse event (AE). Solicited local (injection site pain, erythema, and induration) and systemic (arthralgia, chills, fatigue, malaise, myalgia, fever, headache) AEs were recorded on diary cards by study participants for 7 days post-each vaccination. Solicited AEs after the bacterial challenge were diarrhoea, abdominal pain, abdominal cramps, gas, anorexia, nausea, headache, myalgia, malaise, arthralgia, fever, fatigue, and vomiting, collected by the investigator up to 8 days post-challenge. Unsolicited AEs were collected up to 28 days post-each injection and 28 days post-challenge. All AEs were graded for severity on a scale from 0 to 3 or 4 by the investigator (**Table S1**). Serious AEs (SAEs) were collected during the entire study period. Causal relationship of unsolicited AEs or SAEs to 1790GAHB vaccine/placebo or challenge inoculum was also assessed by the investigator. All study participants with a neutropenia (ANC < 1800 cells/µL) had additional blood draws on a weekly basis until resolution of neutropenia. Neutropenia was diagnosed by laboratory testing for complete blood count and was defined as a decrease of neutrophil count asymptomatically or symptomatically. Symptomatic neutropenia (according to investigator assessment) was considered as AE of special interest and collected during the entire study duration. The number and percentage of participants with AEs were calculated with their exact 95% CI.

**Enzyme-linked immunosorbent assay**

Antibodies elicited to *S. sonnei* LPS were assessed by ELISA using *S. sonnei* lipopolysaccharide (LPS) as plate coating antigen. Nunc Maxisorp round-bottom 96-well plates were coated over night at 2–8°C with 0.5 μg/mL LPS purified from *S. sonnei* strain NVGH1859 by phenol extraction in phosphate-buffered saline (PBS). The plates were blocked for 1h with 5% milk in PBS and subsequently washed three times with PBS containing 0.05% Tween 20 (PBST). Human sera were diluted 1:100 and 1:4000 in 5% milk in PBS and analyzed in triplicate. The samples were tested in comparison to previously established and calibrated anti-*S. sonnei* LPS human standard serum NVGH2863 (pooled serum from 1790GAHB Phase 1 clinical trial in France[^14^](#_ENREF_14)) included in a duplicate series of dilutions on each of the plates. Diluted sera were incubated for 2h in the ELISA plates. After incubation, the plates were washed three times as above. Bound antibodies were detected by 1h incubation with goat anti-human immunoglobulin G conjugated to alkaline phosphatase (Sigma Aldrich, A3187), diluted in PBST 0.1% bovine serum albumin, followed by three washing steps and a color reaction with p-nitrophenyl phosphate substrate (Sigmafast, N2770). After 1h, absorbance (optical density, OD) was measured at 405 nm and 490 nm and the OD405nm–490nm (to subtract the background absorbance) was calculated. Results are expressed in ELISA units determined relative to the standard serum curve. One ELISA unit equals the reciprocal of the dilution of the standard serum giving an OD405nm–490nm of 0.4–0.6 in the assay. Two controls were included in each plate and the interpolated value in EU/mL had to fall in the accepted range to validate each plate. Lower limit of quantification of the assay was of 22 EU/mL.

# Table S1. Grading of solicited adverse events

|  | **Grading** | | | | |
| --- | --- | --- | --- | --- | --- |
|  | **Absent** | **Mild** | **Moderate** | **Severe** | |
|  | **0** | **1** | **2** | **3** | **4** |
| *SOLICITED ADVERSE EVENTS* | | | | |  |
| **After 1790GAHB/placebo administration** | | | | |  |
| Pain at injection site | Absent | Easily tolerated | Interferes with normal activity | Prevents normal activity | – |
| Erythema at injection site (diameter) | <25 mm | 25–50 mm | >50–100 mm | >100 mm | – |
| Induration at injection site (diameter) |  |  |  |  |  |
| Headache^#^ | Absent | Easily tolerated | Interferes with normal activity | Prevents normal activity | – |
| Fatigue^#^ |  |  |  |  |  |
| Arthralgia^#^ |  |  |  |  |  |
| Malaise^#^ |  |  |  |  |  |
| Myalgia^#^ |  |  |  |  |  |
| Chills |  |  |  |  |  |
| Fever^#^ (oral) | <38.0°C | ≥38.0–<39.0°C | ≥39.0–<40.0°C | ≥40.0°C | – |
| **After bacterial challenge administration** | | | | |  |
| Nausea | Absent | Mild or transient; maintains reasonable intake | Moderate discomfort; intake decreased significantly; some activity limited | No significant intake and requires medical intervention | Hospitalization required |
| Abdominal cramping | Absent | No interference with daily activities | Some interference with daily activities not requiring medical intervention | Prevents daily activities and requires medical intervention | ER visit or hospitalization |
| Abdominal pain | Absent | Easily tolerated | Interferes with normal activity | Prevents normal activity | – |
| Gas |  |  |  |  |  |
| Anorexia |  |  |  |  |  |
| Vomiting | Absent | One episode within 24-hour period | Two episodes within 24-hour period | Two or more episodes within 24-hour period and requires medical intervention | ER visit or hospitalization for hypotensive shock |
| Diarrhoea* | Absent | 2–3 Grade 3–5 stools (loose or watery) or <400g of Grade 3–5 (loose or watery) stools per 24 hours | 4–5 Grade 3–5 stools (loose or watery) or 400–800g of Grade 3–5 (loose or watery) stools per 24 hours | 6 or more Grade 3–5 stools (loose or watery) or >800g of Grade 3–5 (loose or watery) stools per 24 hours and requires medical intervention | ≥10 Grade 3–5 loose stools or ≥1000g of Grade 3–5 stools per 24 hours |
| *UNSOLICITED ADVERSE EVENTS* | – | Transient with no limitation in normal daily activity | Some limitation in normal daily activity | Unable to perform normal daily activity | |

^#^Same grading is used for events present both post-vaccination and post-challenge. *The end of a diarrheal episode occurs when an individual dose not pass any Grade 3–5 stool within 24 hours. ER, emergency room.

# Table S2. Participants reporting unsolicited adverse events during the 28-day post-vaccination period (any dose) (unsolicited safety set)

| **System Organ Class/ Preferred Term** | **1790GAHB group**  **N=36** | **Placebo group**  **N=34** |
| --- | --- | --- |
| At least one unsolicited adverse event, n (%) | 18 (50·0%) | 13 (38·2%) |
| Gastrointestinal disorders | 2 (5·6%) | 3 (8·8%) |
| Diarrhoea | 1 (2·8%) | 0 (0·0%) |
| Nausea | 0 (0·0%) | 2 (5·9%) |
| Toothache | 1 (2·8%) | 1 (2·9%) |
| Vomiting | 0 (0·0%) | 2 (5·9%) |
| General disorders and administration site conditions | 1 (2·8%) | 0 (0·0%) |
| Injection site pain | 1 (2·8%) | 0 (0·0%) |
| Infections and infestations | 7 (19·4%) | 2 (5·9%) |
| Cellulitis | 1 (2·8%) | 0 (0·0%) |
| Influenza | 1 (2·8%) | 0 (0·0%) |
| Laryngitis | 0 (0·0%) | 1 (2·9%) |
| Sinusitis | 0 (0·0%) | 1 (2·9%) |
| Tooth abscess | 1 (2·8%) | 0 (0·0%) |
| Upper respiratory tract infection | 4 (11·1%) | 0 (0·0%) |
| Injury, poisoning and procedural complications | 3 (8·3%) | 4 (11·8%) |
| Burns third degree | 1 (2·8%) | 0 (0·0%) |
| Limb injury | 0 (0·0%) | 2 (5·9%) |
| Skin abrasion | 0 (0·0%) | 2 (5·9%) |
| Skin laceration | 1 (2·8%) | 0 (0·0%) |
| Thermal burn | 1 (2·8%) | 0 (0·0%) |
| Nervous system disorders | 2 (5·6%) | 1 (2·9%) |
| Dizziness | 0 (0·0%) | 1 (2·9%) |
| Paraesthesia | 1 (2·8%) | 0 (0·0%) |
| Presyncope | 1 (2·8%) | 0 (0·0%) |
| Musculoskeletal and connective tissue disorders | 3 (8·3%) | 1 (2·9%) |
| Arthralgia | 1 (2·8%) | 0 (0·0%) |
| Muscle spasms | 1 (2·8%) | 1 (2·9%) |
| Pain in extremity | 1 (2·8%) | 0 (0·0%) |
| Respiratory, thoracic and mediastinal disorders | 1 (2·8%) | 2 (5·9%) |
| Epistaxis | 0 (0·0%) | 1 (2·9%) |
| Nasal congestion | 0 (0·0%) | 1 (2·9%) |
| Sinus congestion | 1 (2·8%) | 0 (0·0%) |
| Investigations | 2 (5·6%) | 4 (11·8%) |
| Neutrophil count decreased | 2 (5·6%) | 4 (11·8%) |

N, number of participants in each group; n (%), number (%) of participants reporting the adverse event.

# Table S3. Participants reporting unsolicited adverse events during the 28-day post-challenge (unsolicited safety set)

| **System Organ Class/ Preferred Term** | **1790GAHB group**  **N=33** | **Placebo group**  **N=29** |
| --- | --- | --- |
| At least one unsolicited adverse event, n (%) | 20 (60·6%) | 17 (58·6%) |
| Blood and lymphatic system disorders | 1 (3·0%) | 1 (3·4%) |
| Lymph node pain | 1 (3·0%) | 0 (0·0%) |
| Lymphadenopathy | 0 (0·0%) | 1 (3·4%) |
| Gastrointestinal disorders | 3 (9·1%) | 6 (20·7%) |
| Aphthous ulcer | 0 (0·0%) | 1 (3·4%) |
| Diarrhoea haemorrhagic | 1 (3·0%) | 0 (0·0%) |
| Dyspepsia | 1 (3·0%) | 4 (13·8%) |
| Irritable bowel syndrome | 1 (3·0%) | 0 (0·0%) |
| Proctalgia | 0 (0·0%) | 2 (6·9%) |
| Ear and labyrinth disorders | 1 (3·0%) | 0 (0·0%) |
| Ear congestion | 1 (3·0%) | 0 (0·0%) |
| Ear pain | 1 (3·0%) | 0 (0·0%) |
| General disorders and administration site conditions | 9 (27·3%) | 6 (20·7%) |
| Chills | 7 (21·2%) | 3 (10·3%) |
| Fatigue | 4 (12·1%) | 4 (13·8%) |
| Infections and infestations | 2 (6·1%) | 1 (3·4%) |
| Fungal infection | 1 (3·0%) | 0 (0·0%) |
| Upper respiratory tract infection | 1 (3·0%) | 1 (3·4%) |
| Injury, poisoning and procedural complications | 7 (21·2%) | 6 (20·7%) |
| Anal injury | 2 (6·1%) | 2 (6·9%) |
| Arthropod bite | 1 (3·0%) | 0 (0·0%) |
| Muscle pain | 0 (0·0%) | 1 (3·4%) |
| Skin abrasion | 3 (9·1%) | 3 (10·3%) |
| Skin laceration | 1 (3·0%) | 0 (0·0%) |
| Psychiatric disorders | 3 (9·1%) | 3 (10·3%) |
| Insomnia | 3 (9·1%) | 3 (10·3%) |
| Musculoskeletal and connective tissue disorders | 2 (6·1%) | 4 (13·8%) |
| Arthralgia | 1 (3·0%) | 0 (0·0%) |
| Back pain | 1 (3·0%) | 4 (13·8%) |
| Pain in extremity | 0 (0·0%) | 1 (3·4%) |
| Respiratory, thoracic and mediastinal disorders | 2 (6·1%) | 0 (0·0%) |
| Nasal congestion | 1 (3·0%) | 0 (0·0%) |
| Oropharyngeal pain | 1 (3·0%) | 0 (0·0%) |
| Skin and subcutaneous tissue disorders | 3 (9·1%) | 1 (3·4%) |
| Pruritus | 1 (3·0%) | 0 (0·0%) |
| Rash | 1 (3·0%) | 0 (0·0%) |
| Skin irritation | 1 (3·0%) | 1 (3·4%) |
| Reproductive system and breast disorders | 0 (0·0%) | 1 (3·4%) |
| Dysmenorrhoea | 0 (0·0%) | 1 (3·4%) |
| Vulvovaginal pruritus | 0 (0·0%) | 1 (3·4%) |
| Investigations | 0 (0·0%) | 1 (3·4%) |
| Neutrophil count decreased | 0 (0·0%) | 1 (3·4%) |

N, number of participants in each group; n (%), number (%) of participants reporting the adverse event.

# Table S4. Percentage of participants with anti-*S. sonnei* LPS serum IgG ≥268 EU/mL, geometric mean concentrations and geometric mean ratios by time point (per-protocol set)

|  | **% ≥268 EU/mL (95% CI)** | |  | **GMC [EU/mL] (95% CI)** | |  |  | **GMR (95% CI)** | |
| --- | --- | --- | --- | --- | --- | --- | --- | --- | --- |
|  | **1790GAHB group** | **Placebo group** |  | **1790GAHB group** | **Placebo group** |  | **Ratio** | **1790GAHB group** | **Placebo group** |
| **Participants who did not develop shigellosis (primary case definition); N=17 [1790GAHB group] and N*=16 [Placebo group]** | | | | | | | | | |
| D1 | 47 (23; 72) | 31 (11; 59) |  | 168·06 (82·42; 342·65) | 194·45 (125·26; 301·87) |  |  | - | - |
| D8 | 65 (38; 86) | 25 (7; 52) |  | 418·46 (187·44; 934·21) | 189·93 (122·28; 295·00) |  | D8/D1 | 2·49 (1·43; 4·33) | 0·98 (0·91; 1·05) |
| D29 | 76 (50; 93) | 25 (7; 52) |  | 749·80 (342·41; 1641·89) | 186·78 (116·78; 298·73) |  | D29/D1 | 4·46 (2·66; 7·48) | 0·96 (0·89; 1·04) |
| D36 | 71 (44; 90) | 19 (4; 46) |  | 849·25 (397·67; 1813·60) | 181·24 (116·33; 282·36) |  | D36/D1 | 5·05 (3·10; 8·24) | 0·93 (0·86; 1·01) |
| D57 | 76 (50; 93) | 25 (7; 52) |  | 801·18 (371·55; 1727·60) | 199·36 (128·38; 309·58) |  | D57/D1 | 4·77 (3·04; 7·47) | 1·03 (0·93; 1·13) |
| D64 | 71 (44; 90) | 31 (11; 59) |  | 836·68 (396·48; 1765·62) | 213·31 (135·18; 336·61) |  | D64/D57 | 1·04 (0·95; 1·15) | 1·07 (0·97; 1·18) |
| D85 | 82 (57; 96) | 57 (29; 82) |  | 778·87 (372·95; 1626·57) | 465·06 (224·80; 962·11) |  | D85/D57 | 0·97 (0·81; 1·16) | 2·27 (1·32; 3·92) |
| **Participants who developed shigellosis (primary case definition); N*=15 [1790GAHB group] and N=12 [Placebo group]** | | | | | | | | | |
| D1 | 13 (2; 40) | 8 (0; 38) |  | 52·75 (29·39; 94·69) | 76·63 (29·69; 197·83) |  |  | - | - |
| D8 | 27 (8; 55) | 8 (0; 38) |  | 113·76 (58·01; 223·08) | 88·37 (38·68; 201·88) |  | D8/D1 | 2·16 (1·38; 3·37) | 1·15 (0·92; 1·44) |
| D29 | 60 (32; 84) | 17 (2; 48) |  | 321·15 (132·84; 776·40) | 84·71 (32·92; 217·95) |  | D29/D1 | 6·09 (3·19; 11·61) | 1·11 (0·85; 1·43) |
| D36 | 60 (32; 84) | 17 (2; 48) |  | 347·74 (156·23; 773·99) | 87·45 (34·83; 219·55) |  | D36/D1 | 6·59 (3·64; 11·93) | 1·14 (0·98; 1·33) |
| D57 | 60 (32; 84) | 17 (2; 48) |  | 301·19 (139·17; 651·83) | 89·49 (36·92; 216·89) |  | D57/D1 | 5·71 (3·33; 9·80) | 1·17 (0·96; 1·43) |
| D64 | 67 (38; 88) | 50 (21; 79) |  | 356·57 (144·05; 882·62) | 176·18 (71·34; 435·06) |  | D64/D57 | 1·18 (0·83; 1·69) | 1·97 (1·17; 3·33) |
| D85 | 86 (57; 98) | 75 (43; 95) |  | 1509·89 (664·54; 3430·58) | 1281·12 (371·41; 4419·02) |  | D85/D57 | 4·94 (2·85; 8·54) | 14·32 (5·76; 35·56) |

LPS, lipopolysaccharide; IgG, immunoglobulin G; EU, enzyme-linked immunosorbent assay units; N, number of participants; GMC, geometric mean concentration; GMR, within-subjects geometric mean ratio; CI; confidence interval; D, day. *N=14 at D85.

# Table S5. Serum bactericidal activity geometric mean titres (GMT) and geometric mean ratios (GMR), by time point (per-protocol set)

|  | **GMT (95% CI)** | | |  |  | | | **GMR (95% CI)** | | |  |
| --- | --- | --- | --- | --- | --- | --- | --- | --- | --- | --- | --- |
|  |  | **1790GAHB group** | **Placebo group** | | |  | **Ratio** | | **1790GAHB group** | **Placebo group** | |
| **Overall population; N=32 [1790GAHB group] and N=28 [Placebo group]** | | | | | | | | | | | |
| D1 |  | 81·95 (57·27; 117·26) | 63·52 (48·89; 82·53) | | |  |  | | - | - | |
| D29 |  | 204·65 (113·56; 368·80) | 74·10 (53·49; 102·66) | | |  | D29/D1 | | 2·50 (1·48; 4·20) | 1·17 (0·91; 1·50) | |
| D57 |  | 171·11 (99·57; 294·04) | 89·82 (63·24; 127·57) | | |  | D57/D1 | | 2·09 (1·23; 3·55) | 1·41 (1·07; 1·87) | |
| **Participants who did not develop shigellosis (primary case definition); N=17 [1790GAHB group] and N=16 [Placebo group]** | | | | | | | | | | | |
| D1 |  | 126·74 (67·77; 237·03) | 71·25 (45·30; 112·07) | | |  |  | | - | - | |
| D29 |  | 494·64 (212·90; 1149·23) | 93·11 (53·63; 161·67) | | |  | D29/D1 | | 3·90 (1·64; 9·31) | 1·31 (0·88; 1·95) | |
| D57 |  | 334·78 (152·43; 735·27) | 108·45 (62·15; 189·26) | | |  | D57/D1 | | 2·64 (1·07; 6·51) | 1·52 (0·98; 2·37) | |
| **Participants who developed shigellosis (primary case definition); N=15 [1790GAHB group] and N=12 [Placebo group]** | | | | | | | | | | | |
| D1 |  | 50·00 (50·00; 50·00) | 54·50 (45·08; 65·89) | | |  |  | | - | - | |
| D29 |  | 75·27 (45·60; 124·24) | 54·65 (44·93; 66·47) | | |  | D29/D1 | | 1·51 (0·91; 2·48) | 1·00 (0·75; 1·33) | |
| D57 |  | 79·97 (44·16; 144·83) | 69·85 (46·74; 104·40) | | |  | D57/D1 | | 1·60 (0·88; 2·90) | 1·28 (0·91; 1·81) | |

N, number of participants; CI; confidence interval; D, day; GMT, geometric mean titre; GMR, within-subjects geometric mean ratio.

# Graphical abstract


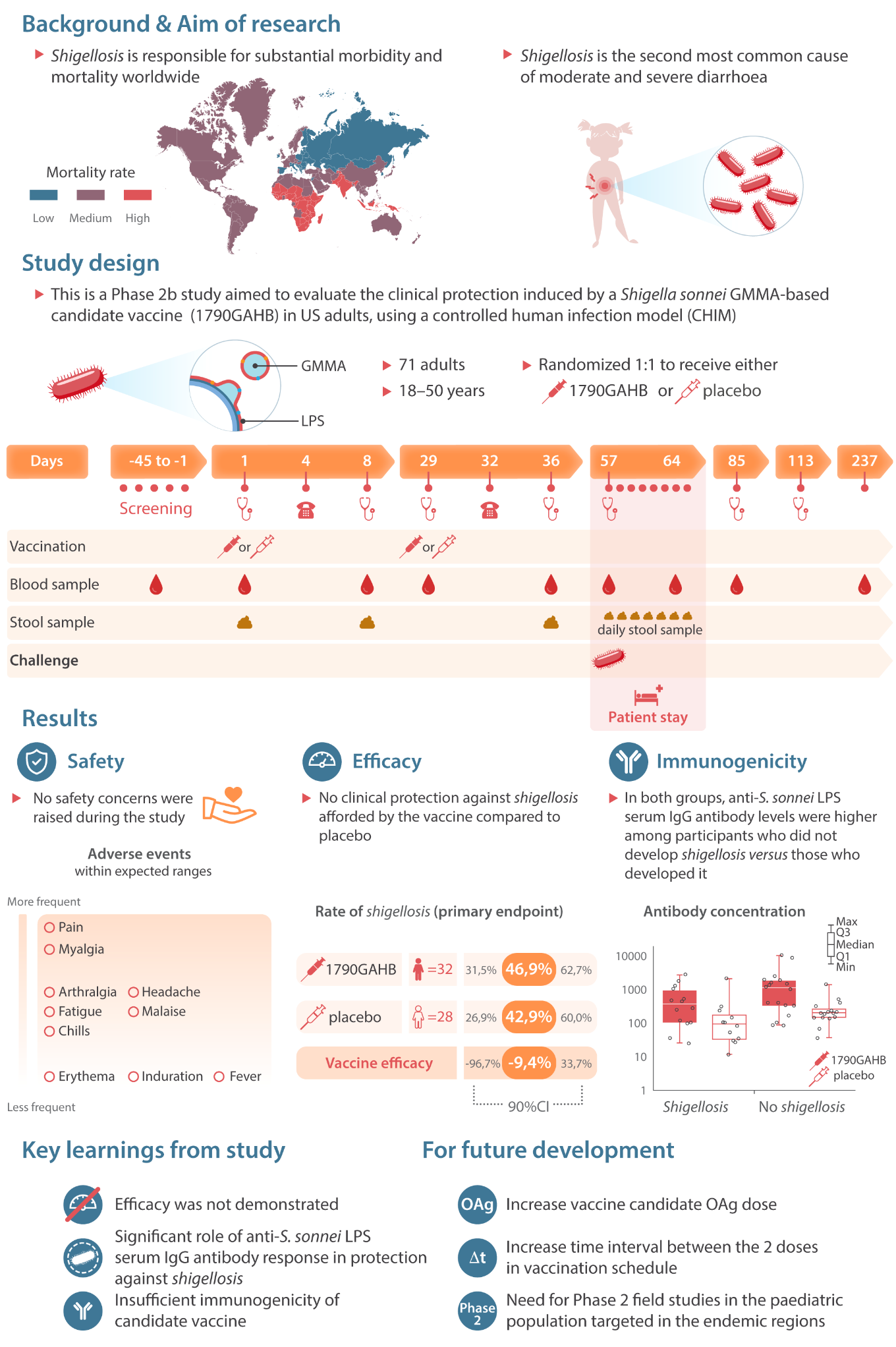


# Figure S1. Plain Language Summary


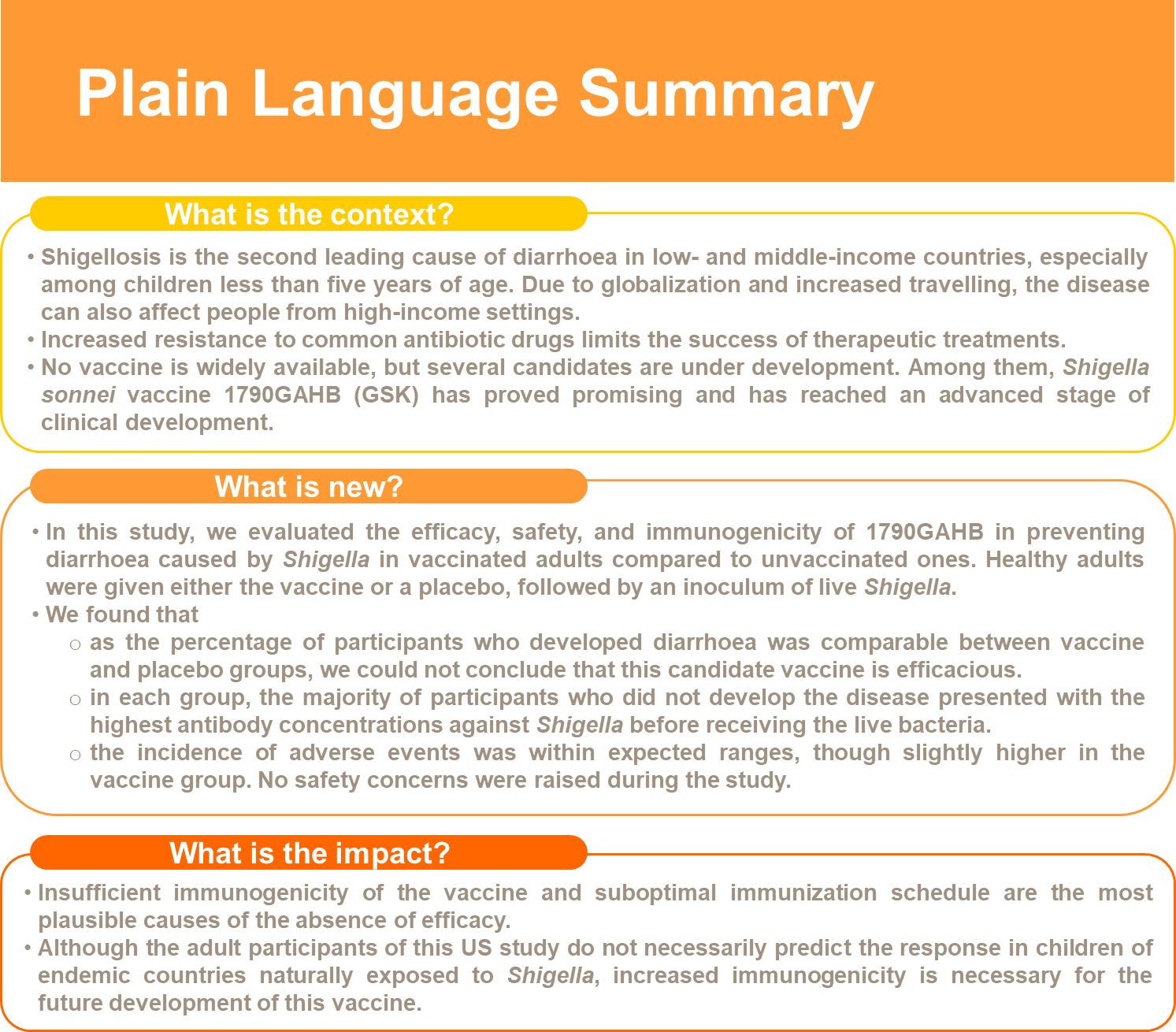


# Figure S2. Anti-*S. sonnei* LPS serum IgG antibody concentration at pre-challenge visit (D57) (per-protocol set)


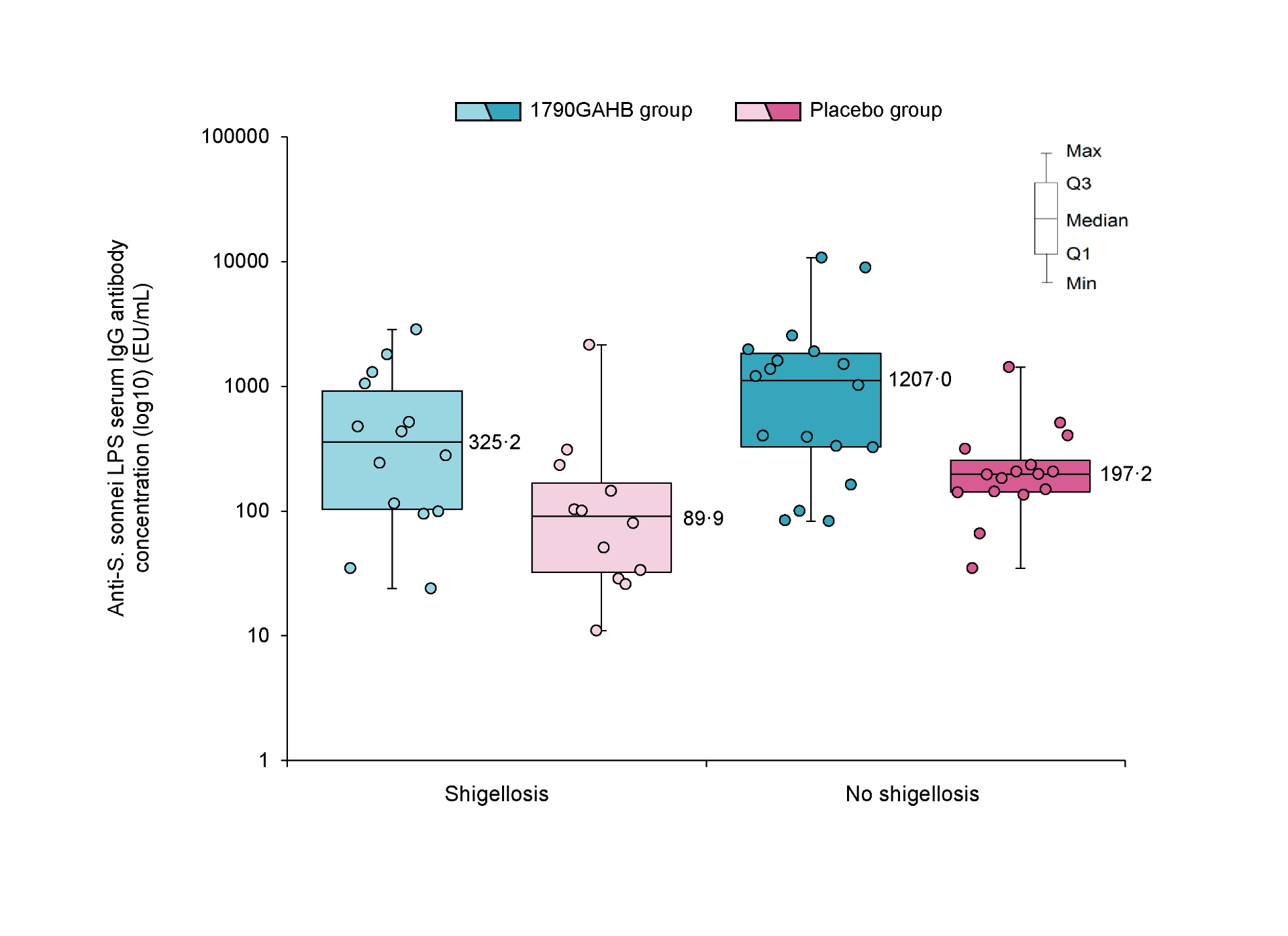


LPS, lipopolysaccharide; IgG, immunoglobulin G; D, day; EU, enzyme-linked immunosorbent assay units; Q1, Q3, quartile 1 and quartile 3.

# Figure S3. Serum bactericidal activity geometric mean titres at pre-challenge visit (D57) (per-protocol set)


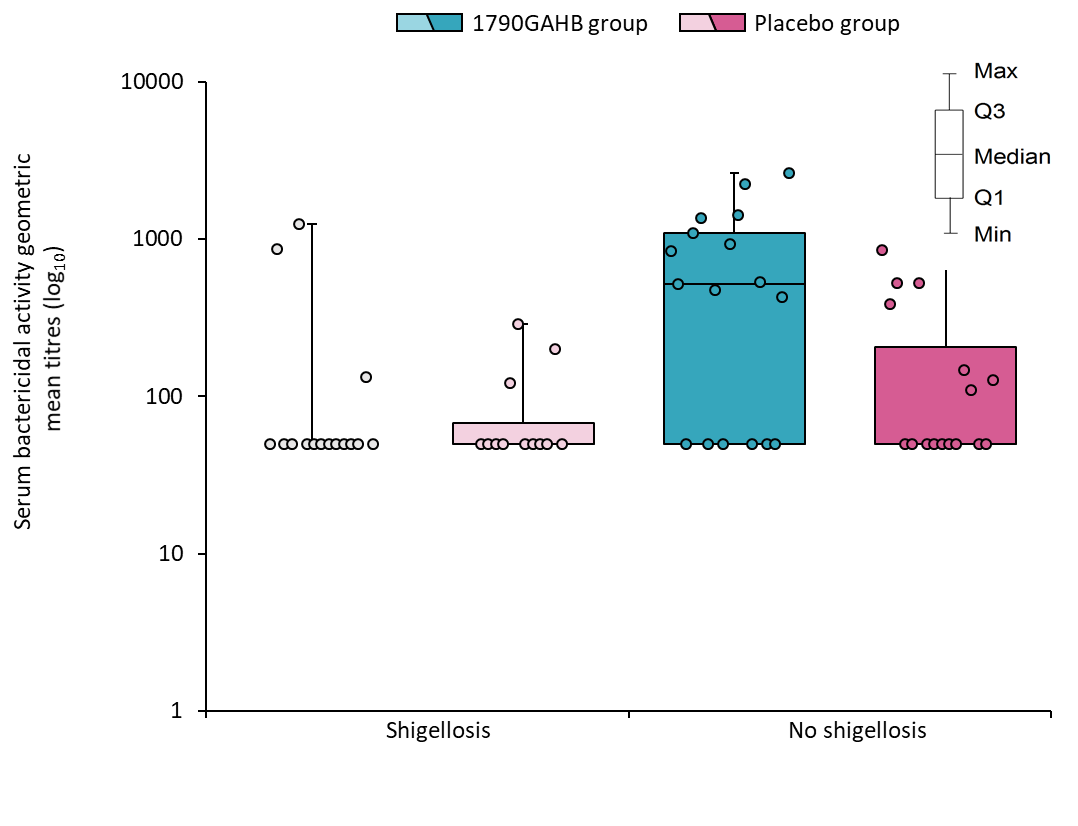


D, day; Q1, Q3, quartile 1 and quartile 3.
